# Searching for new community engagement approaches in the Netherlands: a realist qualitative study

**DOI:** 10.1186/s12889-020-08616-6

**Published:** 2020-04-16

**Authors:** E. De Weger, N. J. E. Van Vooren, H. W. Drewes, K. G. Luijkx, C. A. Baan

**Affiliations:** 1 National Institute for Health and the Environment (RIVM), P.O. Box 1, 3720 BA Bilthoven, The Netherlands; 2 Tilburg University, Tranzo, Tilburg School of Social and Behavioural Sciences, PO Box 90153, 5000 LE Tilburg, The Netherlands

**Keywords:** Community engagement, Citizen engagement, Community participation, Healthcare, Realist evaluation, Decentralisation

## Abstract

**Background:**

Community engagement is increasingly seen as key to improving healthcare systems and to increasing communities’ involvement in the shaping of their own communities. This paper describes how ‘community engagement’ (CE) is understood and being operationalised in the Dutch healthcare system by investigating the CE approaches being implemented in six different regions and by examining engaged citizens’ and professionals’ experiences of those CE approaches.

**Methods:**

For this realist study, interviews and focus groups were held with citizens (16) and professionals (42) involved in CE approaches in the six regions. Additionally, CE-related activities were observed to supplement interview data.

**Results:**

This study shows that citizens and professionals defined and experienced CE differently and that they differed in who they felt had ownership of CE. The CE approaches implemented in community-led initiatives and organisationally-led initiatives varied accordingly. Furthermore, both citizens and professionals were searching for meaningful ways for citizens to have more control over healthcare in their own communities.

**Conclusion:**

CE can be improved by, first of all, developing a shared and overarching vision of what CE should look like, establishing clear roles and remits for organisations and communities, and taking active measures to ensure CE is more inclusive and representative of harder-to-reach groups. At the same time, to help ensure such shared visions do not further entrench power imbalances between citizens and professionals, professionals require training in successful CE approaches.

## Background

Community engagement (CE) is seen as key to the development of citizen-centred and sustainable healthcare systems and to the improvement of citizens’ health and wellbeing [5, 30]. The aim of CE is to involve citizens in the decision-making, planning, designing, governance and delivery of services and policies [30]. CE approaches can range from consultation—citizens have limited power to influence decision-making—to partnership and (shared) leadership—where citizens have decision-making control [5, 31, 39]. The approaches can therefore take many different forms including citizen advisory panels, peer healthcare delivery, or community-led initiatives [30].

Despite the fact that meaningful CE remains challenging to implement or support [2, 27, 42]. community-led initiatives, organisations, and governments are looking to enable citizens to have a more active role in the shaping of services, policies and their own communities. For example, in the Netherlands, neighbourhoods have formed cooperatives to produce their own green energy or to provide each other with informal care [3, 41, 44]. While in Australia, Aboriginal community members have been engaged in redesigning health services to reduce health inequalities [13].

With the concept of a ‘participation society’ [37] the Dutch government emphasised the transition from a ‘classic welfare state’ to a ‘participation society’ requiring all able citizens to take responsibility for their own lives and environments [44]. This transition coincided with the decentralisation of health and care policies and services [9, 38, 46] and with a sharp increase in community-led initiatives which are broadly centred on issues regarding health, care and healthy neighbourhoods [29].

In response to this transition and because successful CE remains difficult to achieve, many organisations in the Netherlands, and internationally, have begun searching for new ways to engage citizens in the development and improvement of health and care policies and services [12, 41]. Previous studies have investigated how individual health and care organisations are engaging citizens (e.g. [11, 15, 34]), described the development of specific CE approaches e.g. [41, 49], or suggested guiding principles for the successful implementation of CE approaches [12]. However, these studies do not provide an overview of how CE is understood or being translated within the health and care sector. For example, it remains unclear how communities and organisations define and experience ‘CE’ or how professionals and citizens are adapting their roles to better suit a ‘participation society’. To provide insights into the current Dutch CE situation, the authors are conducting a four-year multiple case-study investigating how six different regions are developing and implementing their own CE approaches. The first stage of the study consisted of an international rapid realist review (RRR) regarding the barriers and enablers for engaging communities and developed eight guiding principles for the successful implementation of CE. The initial eight guiding principles are: (a) ensure staff provide supportive and facilitative leadership to citizens; (b) foster a safe and trusting environment enabling citizens to provide input; (c) ensure citizens’ early involvement; (d) share decision-making and governance control with citizens; (e) acknowledge and address citizens’ experiences of power imbalances between citizens and professionals; (f) invest in citizens who feel they lack the skills and confidence to engage; (g) create quick and tangible wins; (h) take into account both citizens’ and organisations’ motivations [12].

Building on from the RRR by using the guiding principles as initial programme theories, and as the basis for the analytical framework, this paper describes the second stage of the study, which explored the underlying contextual factors and mechanisms explaining how CE is being developed and experienced in six different regions in the Netherlands. The study explored the following research questions:

1. What CE approaches are being developed and implemented in the six regions and how do these compare to professionals’ and citizens’ definitions and underlying expectations of CE?
2. What are the underlying mechanisms explaining engaged citizens’ and professionals’ experiences of CE approaches (including enablers and barriers)?

## Methods

For this study, qualitative interviews and focus groups with citizens and professionals were held and the realist methodology was applied. A key aspect of the realist methodology is the notion that interventions work differently in different contexts. This means that CE approaches may be successful in some contexts and not in others, because the ‘mechanisms’ needed for success are triggered to a different extent and lead to different outcomes [17] *See* Table 1*for definitions of key realist concepts)*. Continuing on from the RRR [12], the guiding principles were used to examine which enabling and constraining factors were triggered within the contexts of the six different regions; thus refining the guiding principles. Furthermore, this study was undertaken in consultation with a local reference panel, consisting of the six regions’ stakeholders, including professionals, citizens, and Patient Public Involvement (PPI) organisations who will be further developing and implementing the CE approaches.

**Table 1:** CE-oriented definitions of realist concepts

*Intervention*	Refers to interventions’ implemented activities, strategies and resources [21], e.g.: citizen advisory panel meetings, or neighbourhood organized workshops.
*Context*	Pertains to the backdrop of an intervention and includes the pre-existing organizational structures, cultural norms of the community, the nature and scope of pre-existing networks and geographic location effects [18, 25].
*Mechanism*	‘Mechanism’ does not refer to the intentional resources offered or strategies implemented within an intervention. Instead, it refers to what ‘triggers’ participants to want to participate or not in an intervention. Mechanisms usually relate to cognitive, emotional or behavioural responses to intervention resources or strategies [18]. Mechanisms are usually hidden, sensitive to variations in context, and generate outcomes [1]: e.g. citizens feeling more empowered due to learning opportunities.
*Outcome*	Refers to intended, unintended, or unexpected intervention outcomes ([18]; e.g. sustainability, quality and integration of services (macro), citizens’ level of involvement in health and care services (e.g. in designing services) (meso), citizens’ health and wellbeing outcomes (micro).
*Context-M-echanism-Outcome configuration (CMO)*	To understand how certain contextual factors shape or trigger the mechanism, causal links are expressed through ‘Context-mechanism-outcome configurations’ (CMOs). Formulating and refining CMOs is largely how researchers analyse data in a realist evaluation as it allows for a deeper understanding of which (aspects of) of interventions work, for whom, under what circumstances and to what extent [48]. CMOs are also used to generate or refine programme theories, which in turn help shape the final product of an evaluation (e.g. recommendations). CMOs are also used to generate or refine programme theories.
*Programme theory*	Is a hypothesis about how a programme or programme component may or may not work, under what contexts and with what outcomes [32]. In this study, the guiding principles [12], which can be seen as action-oriented programme theories, were used as initial programme theories.

*Sources:* [12, 35]

Data were collected between November 2017 and March 2018. A semi-structured interview guide, informed by the guiding principles, was used to anchor the interview process *(available upon request)*. HD and EdW conducted the interviews and focus groups. All interviews and focus groups were held when and where professionals or citizens would normally meet in order to reduce the burden on participants (e.g. their offices or local meeting points). All interviews and focus groups were recorded and transcribed to aid data analysis with participants’ permission. The interview data were supplemented and triangulated by fieldnotes taken by the authors during the observations of CE-related activities.

### Study sample & recruitment strategy and data collection

For this study, purposive sampling and snowball sampling [26] were used to ensure key CE approaches operating on a participation level within the regions were sufficiently reflected within the sample. The authors used Rowe & Frewer’s [39] typology of participation to ensure CE approaches included in the sample not only operated on the ‘public communication level’ (whereby citizens receive information from organisations), or on the ‘public consultation level’ (whereby citizens provide information to organisations), but also on the ‘public participation level’ whereby citizens are actively engaged in dialogue with organisations. To consider contextual differences, professionals from different sectors and organisations who had an active role in CE approaches were interviewed as well as citizens engaged in different types of CE approaches (both community-led and organisationally led initiatives). The professionals and citizens were recruited through the local reference panel members’ networks. All approached potential participants agreed to take part in a focus group or interview and had signed consent forms. Ultimately, a total of eight focus groups (each one lasting about 2 hours) and four interviews (each one lasting about 1 hour) were held with 42 professionals and 16 citizens (total: 58 participants) *(See* Table 2*)*. Interviews were conducted until the authors agreed the point of data saturation was reached; when no new themes emerged and when there was a high rate of recurrence of responses [14]. Additionally, for data triangulation a total of six observations were held in five of the six regions *(See* Additional file 1*).* Finally, the authors held a workshop to present and discuss the study’s findings to the local reference panel. The panel discussed whether the findings had face validity within their own local contexts and confirmed data saturation had been reached.

**Table 2:** Summary of regions’ implemented CE approaches

**Region A contextual factors**: *Rural area including two municipalities which receive funding & support for addressing (health) inequalities, 106,500 residents*	
**Consultation/Communication level**	**Participation level**
• Region’s Public Health organisation, commissioned by a local municipality, conducted focus groups and informal dinner events with residents discussing their experiences of living in the area *(municipality-wide & initiated by municipality)* • ‘Living rooms’ across the region set up to enable organisations and residents to discuss and address urgent healthcare issues together *(region-wide & initiated by health and business consultancies)*	Online Platform enabled all residents and professionals within the region to share and discuss ideas to improve the region’s healthcare system. Because the Platform was resource intensive, it has gone off line *(region-wide & initiated by region’s joined governance board)*
**Region B contextual factors**: *Mixed rural & urban area including two municipalities which receive funding & support for addressing (health) inequalities, 270,000 residents*	
**Consultation/Communication level**	**Participation level**
	• ‘WeHelp’ online platform enabling residents to ask for and provide each other with informal help, e.g. mowing each other’s lawn, social visits *(region-wide & initiated by citizens)* • Local resident and the local PPI organisation were members of health and care system’s joined governance board *(region-wide & initiated by joined governance board)*
**Region C contextual factors**: *Rural area including one municipality, 27,500 residents*	
**Consultation/Communication level**	**Participation level**
Municipality professionals establishing closer relationships with residents, local sports clubs, and village councils in order to promote and improve CE *(municipality-wide & initiated by municipality)*	Municipality engaging schools, parents and students in developing municipality’s youth policy *(municipality-wide & initiated by municipality)*
**Region D contextual factors**: *Rural area including eight municipalities, 110,000 residents*	
**Consultation/Communication level**	**Participation level**
• Conducted patient satisfaction surveys for general practices (GPs) as part of a new quality improvement system whereby practices would be monitored against the steps being undertaken to improve areas highlighted in the survey (making GPs more accountable to patients) *(region-wide & initiated by primary care group)* • Community-led initiative kicked-off with volunteers going door-to-door with a ‘village diary’ asking residents to write something about their village, e.g. what they liked about the village, what amenities were missing. The diary informed initiative’s priorities *(community-wide & initiated by citizens)* • Health promotion activities *(region-wide & initiated by network of health and care professionals)* • PPI organisation held workshops for municipality and citizen-led initiatives to help them improve their relationship and collaboration *(municipality-wide & initiated by PPI organisation)*	• Primary care group’s client council *(organisation-wide & initiated by primary care group)* • Initiative was set up when village’s only GP retired. The initiative set up a multi-disciplinary medical centre, a free library, a shared neighbourhood-allotment, and organised social activities *(community-wide & initiated by citizens)* • Resident village support worker maintained close links with the community and ensured residents’ health and care needs were addressed (whenever possible by residents themselves and otherwise, by the municipality) *(community-wide & initiated by citizens)*
**Region E contextual factors:** *Rural area including three municipalities, 120,000 residents*	
**Consultation/Communication level**	**Participation level**
• ‘Self-care for me’ website enabled local residents to score their own health. Local municipalities were hoping to get local businesses involved to set up ‘fun challenges’ improving residents’ health *(region-wide & initiated by municipalities)*. • Annual policyholder health promoting events and workshops *(region-wide & initiated by joined governance board & policyholders)* • To improve children’s health, local municipalities were establishing closer relationships with community-led initiatives, sports clubs *(region-wide & initiated by municipality)*	The biggest insurance companies, local municipalities, and health and care providers had set up Policyholder Cooperative to ensure policyholders could have a say in which services should be included within the insurance package and to help shape the local healthcare system. Five policyholders represented the Cooperative *(region-wide & initiated by joined governance board)*.
**Region F contextual factors** *Suburban area including one municipality, 41,000 residents*	
**Consultation/Communication level**	**Participation level**
Health promotion workshops and apps *(municipality-wide & initiated by citizens)*	The initiative designed and implemented health-promotion projects, activities and workshops (e.g. implementing benches along walking paths) *(municipality-wide & initiated by citizens)*

### Data analysis

Applying the realist evaluation approach, the authors constructed context-mechanism-outcome configurations (CMOs) from each interview transcript, in order to examine what works, for whom, under what circumstances, why and how [33]. Interviews were thus analysed using CMOs, which were drafted and placed into Excel by EdW, and refined and confirmed by NvV before being discussed by all authors. To aid the authors during the data analysis process and to ensure consistency and transparency, the authors applied the same CE-oriented definitions of ‘interventions’, ‘contexts’, ‘mechanisms’, and ‘outcomes’*(See* Table 1*)*. The CMOs were drafted in order to explain (a) why some CE interventions in the six regions were perceived as successful, or not; (b) to explain participants’ perceptions and experiences. After drafting the CMOs, the authors thematically clustered the configurations based on the mechanisms in those configurations. The clustering followed a sequential and iterative process:

- CMOs were either clustered in one of the eight guiding principles, or if none of the principles were the right fit, were grouped together in a separate pile labelled ‘additional themes’;
- The authors discussed the ‘additional themes’ CMOs and upon review, clustered some of the CMOs in one of the eight original themes. The other CMOs were again thematically analysed, reviewed and discussed. The last remaining ‘additional’ CMOs generated an additional guiding principle *(See results section)*;
- The final draft of the clustered CMOs was shared with the other authors to confirm and refine the guiding principles.

## Results

The following section first provides a brief overview of CE approaches operational in the six regions at the time of interviewing *(see* Table 2*)*, followed by a description of citizens’ and professionals’ definitions and underlying expectations of CE. Then, per guiding principle, citizens’ and professionals’ experiences, including enablers and barriers, will be described separately, with some examples of individual CMOs underpinning the results *(*Additional file 2*includes further CMO examples)*.

### Implemented CE approaches

While some organisations within the regions had implemented CE approaches at the ‘participation’ level *(See* Table 2*)*, most approaches and underlying activities could be classified at the ‘consultation’, or ‘communication’ level. The types of CE approaches varied accordingly. Firstly, regions had focused their consultation-level activities on receiving residents’ feedback regarding specific topics or policies and had conducted focus groups or surveys to collect their input. For example, in Region A, professionals had held focus groups with elderly residents to discuss what they felt their future health and care needs would be and were intending to use this input to adjust local policies and services (without further citizen-involvement). Secondly, at the communication level, many regions had implemented apps and websites, or had held workshops to promote healthy lifestyles. For example, in Region D, a network of local health and care organisations held a series of workshops within local communities (e.g. in libraries) about topics related to healthy lifestyles, e.g. how to have a good night’s rest. Finally, most organisations within the six regions were yet to successfully shape and implement participation-level approaches. Within each region, traditional CE-initiatives were operational, like organisations’ client councils and village councils. However, only a few municipalities within Regions C, D and E, were actively seeking to establish closer working relationships with these councils to ensure councils (and by extension local residents) were more fully included in the municipalities’ CE approach. Furthermore, the participation-level initiatives perceived as more successful had originated from citizens themselves, like Region F and one of the community-led initiatives in region D.

### Citizens’ and professionals’ CE definitions and underlying expectations

The above highlighted situation—that most CE approaches instigated by professionals could be classified at the consultation- or communication-level, and that many participation-level interventions were initiated by communities—is in line with how professionals and citizens defined ‘community engagement’. Most professionals felt CE consisted of two different approaches. The first approach, led by organisations, allowed professionals to discuss together with citizens, which policies and services should be implemented within local neighbourhoods however, accountability and decision-making still rested with organisations.

Community-led initiatives were seen as the second CE approach, whereby citizens themselves are driven to tackle problems that they experience within their own communities and organisations have a supportive role. There was some overlap between citizens’ and professionals’ definition of community-led initiatives, however professionals and citizens differed in who they felt had ownership of CE. Professionals saw CE as something that organisations can initiate and have ownership of (especially with CE interventions), while citizens saw CE more as a movement, which can only come from communities themselves.

### Experiences of CE approaches

Citizens and professionals had started shaping and implementing CE approaches, but felt that further improvements were necessary to ensure more meaningful engagement with a wider range of citizens. Using the guiding principles as programme theories, the section below first briefly describes the initial programme theories [12], and afterwards examines citizens’ and professionals’ experiences of the implemented CE approaches and to what extent enabling factors were triggered within the contexts of the CE approaches. Thus further refining the guiding principles. Examples of individual CMOs underpinning the results will be included *(See* Additional file 2*for summary of CMOs)*.

### Ensure staff provide supportive and facilitative leadership to citizens

The first guiding principle indicates that organisations should use a supportive and facilitative leadership style that supports citizens in their roles without being directive or restrictive [7, 12, 13, 43, 49]. However, in this study, professionals described how they did not yet have the required skills to adopt a more facilitative, instead of a directive, leadership style. Furthermore, both citizens and professionals lacked a clear leadership figure and experienced this as an important barrier to the improvement of CE approaches.

#### Citizens

For most citizens in our study, a key barrier to their engagement was that they experienced either a lack of support from the organisation they were involved in or lacked a key figure within their own initiative who could take on the supportive leadership role. Of the interviewed citizens, only the community-led initiative in Region D felt supported. They described how most municipalities take a one-size-fits-all approach to CE and provide initiatives with the same support *(context)*. However, their own municipality believed community-led initiatives are best at identifying their own communities’ needs and therefore felt comfortable providing different support to align with communities’ varying contexts *(mechanism)*. This different CE approach has enabled the municipality to provide more tailored support to the community-led initiatives, which citizens respect and appreciate *(outcome)*.

#### Professionals

Professionals described that traditionally, organisations have ownership and control of CE interventions and that they were currently looking for new leadership styles to enable citizens to take ownership instead. For example, professionals in Region C explained that CE would be more successful (and enable services and policies to be better tailored to citizens’ needs) if civil servants ‘gave up ownership’ of CE interventions. However, they thought this was often ‘scary’ for civil servants, as they felt ultimately accountable for interventions’ outcomes to the local council. Reversely, Region A interviewees described that the Online Platform had failed because professionals exclusively held the leadership role. The Platform had been developed and implemented without any input from citizens and was based on services and policies which were already available within the healthcare system, rather than what citizens felt they needed *(context)*. Hence, citizens did not see how the Platform addressed their priorities and felt no connection to it *(mechanism)*. Once operational, the Platform was almost exclusively used by the professionals *(outcome)*.

### Foster a safe and trusting environment enabling citizens to provide input

This principle highlights that creating forums where citizens feel comfortable enough to share their ideas and input is important in ensuring CE is successful [11–13, 19, 20, 24, 28, 40, 47]. However, citizens involved in organisationally-led CE interventions experienced this as an important barrier for them. Though professionals understood the importance of creating such environments, most did not have the experience, knowledge or skills to do so.

#### Citizens

Citizens involved in organisationally-led interventions described their experience of feeling out of place, and ‘out-classed’ by professionals’ knowledge and access to resources. For example, one of the citizens in Region D described her experience of being the only citizen involved in large, cross-sectoral and professionally-led meetings. She suspected most professionals only took part because of their organisational agendas *(context)*. She described how professionals had laughed at her during one of the meetings, when she asked what an abbreviation stood for and never answered her question. This made her feel as if the professionals looked down on her and made her feel mistrustful of them *(mechanism)*. Since then she had not provided any input during the meetings *(outcome)*.

#### Professionals

Region B’s PPI organisation highlighted why many organisations struggled to create a safe and trusting environment and had a tendency to keep all CE activities formal and within the organisational sphere. They described how organisational structures and processes were not aligned with citizens’ needs. They indicated that many professionals never considered the type of support citizens may require or that engaging in organisational projects and processes can take a lot out of citizens unless citizens’ needs are addressed *(context)*. This can be very demotivating for citizens who do not feel appreciated or listened to *(mechanism)*. It also means that most citizens are prevented from participating in CE interventions, apart from those who are already engaged, who are typically middle-class *(outcome)*. One of the senior-level directors on Region B’s cross-sectoral governance board explained that organisations often do not implement CE interventions because they feel it would be too resource-intensive to create safe and trusting environments *(See* Additional file 1). While many professionals thought CE interventions required significant investment, PPI organisations emphasised that being approachable and creating safe and trusting environments leads to better outcomes.

### Ensure citizens’ early involvement

This principle highlights that citizens should be involved as early as possible, so they can identify and prioritise their own needs [6, 8, 12, 22, 43, 47]. However, the point at which citizens got to be involved differed according to the type and level of CE. For example, citizens involved in organisationally-led interventions and in community-led initiatives had experienced the importance of involving (other) citizens early on. Overall, community-led initiatives were successful in getting citizens involved, especially in shorter projects and highlighted that early involvement helped ensure initiatives remained connected to the wider neighbourhood. Incomparison, professionals struggled to engage citizens during CE interventions’ early stages and felt they did not yet have the skills to connect with citizens and communities earlier on.

#### Citizens

Region E’s Cooperative’s experience clearly highlights why citizens should be involved as early as possible. Professionals had developed and implemented the Cooperative without any policyholder input and only afterwards selected five policyholders to represent the others *(context)*. Because the professionals had created the Cooperative, the representatives struggled to become autonomous from the professionals and felt unable to shape their own independent roles *(mechanism)*. Consequently, at the time of interviewing, the Cooperative were still trying to reach out to other policyholders *(outcome)*. In comparison, citizens in Region D’s community-led initiative had reached most of its original goals andinitiative members wanted to involve their neighbours early-on in setting new priorities *(context)*. The initiatives’ members felt accountable to their neighbours and wanted to stay connected to them *(mechanism)*. They worried the initiative would, otherwise, no longer be representative of the entire village or be able to address their neighbours’ needs (*outcome)*.

#### Professionals

Professionals in Region A and C described that as part of the changing CE approaches; organisations were looking to involve citizens earlier—with the expectation that this would garner broader support for their projects or plans—but that this would require a change in municipalities’ organisational cultures, including professionals’ perceptions of citizens’ input. For example, Region A professionals explained that municipalities hesitated to involve citizens early on, because they were worried citizens would be unable to look ‘beyond their personal interests’ and that the municipality would then have to weigh these often opposing interests and decide which ones were ‘valid enough’ to be included in new policies. Similarly, Region D’s professionals described that current organisational structures—which lack clear points of connection to citizens—and tight project deadlines make it difficult for professionals to approach citizens during earlier stages *(See* Additional file 2*)*.

*“As a professional, you’re often very busy creating ideas in your own head. And then the moment when you can start sharing those ideas, it’s often way easier to share it with your colleague, and not with citizens, because well they’re not as visible, are they?”* (Professional)

### Share decision-making and governance control with citizens

The RRR indicated citizens should be encouraged and supported to take on governance and decision-making roles within CE interventions [6, 8, 12, 13, 20, 22, 24]. Citizens discussed the importance of communities having more control over their own neighbourhoods, but highlighted that to enable communities to take control, the diverse make-up of communities would need to be properly reflected within initiatives and interventions—currently they felt it was mostly white, middle-class citizens who are involved in CE. In contrast, professionals still positioned CE within their own spheres of influence by stating that CE interventions’ development and control should not rest ‘exclusively’ with organisations.

#### Citizens

A lot of citizens felt it was important for a wider-range of citizens to have more control over the shaping of their own neighbourhoods. However, the way citizens experienced and discussed this differed. Some citizens wanted more controlby taking over institutional roles while others felt shared control between a wider-range of citizens would lead to a more inclusive community with better social cohesion *(See* Additional file 2*)*. For example, Region D’s community-led initiative were investigating how the initiative could take on municipal responsibilities (e.g. commissioning of health and care services). However, they felt that the initiative could only do this efficiently if the municipality was willing to relinquish control to the community and at the same time collaborate fully with the initiative. However, initiative members were struggling to get other residents interested in more formal matters and felt it was important not to force residents to take on institutional responsibilities. Ultimately, the initiative was still searching for ways to balance the dynamic of a community-led initiative while at the same time taking over institutional roles so the village could control its own fate. Citizens in other regions felt decision-making and control within CE should be shared with a wider range of citizens, not just the ‘usual suspects’ (who tend to be white and middle-class) to ensure neighbourhoods’ diversity is properly reflected *(See* Additional file 2*)*.

*“Well, I would say I’d like to see more diversity [in CE]. Because that’s how we create our community”* (Citizen)

#### Professionals

Region A and C municipality-professionals perceived that the extent to which municipalities should share (decision-making) control depended on interventions’ initiators. They felt that if municipalities developed the intervention, then professionals would have to set the boundaries and communicate those clearly with involved citizens. However, if citizens are the initiators, municipalities should take on a more facilitative role *(context)*. However, giving up control can be very challenging and counterintuitive, especially for local councillors, who often maintain that community initiatives submit to municipal rules and processes *(mechanism)*. The stringent upholding of municipal rules can be very demotivating for citizens *(mechanism)*, which in turn means fewer initiatives are implemented *(outcome)*.

### Acknowledge and address citizens’ experiences of power imbalances between citizens and professionals

The RRR highlighted that addressing power imbalances between citizens and professionals is crucial to CE interventions’ success but that certain factors contribute to citizens’ relative powerlessness; e.g. organisational structures, hierarchies, decision-making positions resting with organisations [6, 12, 20, 23, 24, 36]. Citizens highlighted that they should hold important roles within CE interventions but also that their input should be recognised and appreciated. Professionals indicated that professionals and citizens should be recognised as equals but for their own unique roles and input. Professionals often saw their own role as an ‘expert’ and citizens’ roles as ‘lay expert’; thus often inadvertently reaffirming the power imbalances. Power imbalances between professionals and citizens were largely influenced by the fact that CE interventions remained within the organisational domain and that professionals, knowingly or not, structured the interventions according to their own organisational needs, processes and structures, thus excluding citizens. Furthermore, citizens often felt undermined by professionals’ limited perceptions and expectations of citizens and described how these limited the scope of their involvement. Such power imbalances not only affected CE interventions, but also community-led initiatives, which often required organisational support or investments.

#### Citizens

All interviewed citizens had experienced power imbalances between themselves and organisations. For example, Region E’s professionals had recruited highly educated policyholders as the Cooperative representatives with the expectation that they could understand and provide input into complex issues related to the regional healthcare system *(context)*. At the same time, the professionals expected the members to be representative of all other citizens in the region, including vulnerable and disadvantaged groups (e.g. lower income groups). The Cooperative experienced professionals’ expectations as contradictory and felt at a loss about addressing either expectation without more support (e.g. in reaching out to other citizens) *(mechanism)*. Because the Cooperative had not fulfilled the professionals’ contradictory expectations, professionals had largely ignored the Cooperative’s input *(outcome)*. While Region D’s community-led initiative’s experience highlighted that organisations’ fragmented structures are often a barrier to community-led initiatives’ more holistic views and approaches *(See* Additional file 2*)*.

#### Professionals

Professionals recognised that there were power imbalances but many downplayed the negative impact of such imbalances. For example, Region A’s professionals glossed over the power imbalances between professionals and citizens by overemphasising power imbalances between participating and non-participating citizens and viewed engaged-citizens as not representative of others *(See* Additional file 2*)*. Similarly, Region B’s PPI organisation indicated that organisations maintained power imbalances by predefining and separating citizens’ and professionals’ roles within CE thus placing their own expertise as ‘professionals’ above that of citizens as ‘lay experts’ *(See* Additional file 2*)*. Finally, Region D PPI professionals described how professionals maintained power imbalances because addressing communities’ needs would fall outside of their organisations’ sphere of influence and control, which would require them to change their remits and processes. The results indicate that many organisations continued to operate from a position of power and that CE interventions’ products and results often do not tie into communities’ needs or expectations.

*“I would say that this suspicion [towards citizens] is connected to this idea of: “I actually know much better than you. I know, as professional, much more than you, citizen”* (Professional).

### Invest in citizens and professionals who feel they lack the required CE skills or resources

The initial guiding principle stated that organisations should offer learning opportunities to citizens who feel they lack the skills, knowhow or confidence to engage. Without being offered the opportunity to learn new skills or capabilities, many citizens may feel unable to fully engage [10–13, 22, 36]. Citizens described their experiences of organisations’ reluctance to invest (financially) in CE interventions if their initiatives did not have a clear business case to underpin their request for support and had experienced a lack of continuity in support. Relatedly, professionals felt that CE was not fully embedded within their organisational cultures and experienced this as a barrier to investing or supporting CE on a longer-term basis. They therefore suggested that not only citizens should be offered learning opportunities, but professionals as well.

#### Citizens

Region E Cooperative members had experienced pressure from organisations to achieve measurable results quickly (especially financially) *(context)* but felt that instead of pressuring citizens, professionals should trust and support them in working towards shared aims *(mechanism)*, as they felt this trust and willingness to invest was key to CE intervenions’ successful outcomes *(outcomes)*. Furthermore, Region D and Region F citizens felt that already engaged citizens should support others to become involved in their own preferred manner; especially disadvantaged or vulnerable citizens *(See* Additional file 2*)*.

#### Professionals

Region B’s PPI professionals suggested that because of this lack of knowledge, professionals maintain existing organisational cultures (of only paying lip service to CE) and remain stuck in their current ways of thinking. In order to change this culture and encourage CE, professionals require additional learning opportunities, which challenge their perceptions of CE and citizens and enables them to think more about citizens’ needs and how organisations could address those (rather than how citizens can help address organisations’ needs). The PPI organisation felt that by investing in professionals’ CE skills, organisations’ CE cultures are gently challenged and professionals’ awareness of enablers and barriers for CE increases.

*Mostly, it’s not people’s unwillingness, but just inexperience. So I would add we need to also train professionals”* (Professionals).

### Take into account both citizens’ and organisations’ motivations

This principle suggests that organisations should enable citizens to participate in projects and interventions that truly interest them, instead of requiring citizens to participate in other projects [11, 12, 16, 22, 23, 34, 40, 45, 47]. Both citizens and professionals wanted to enable communities to get involved in the projects that were of interest to them; but only community-led initiatives and the PPI professionals had any experience in this. Moreover, citizens’ experiences emphasise the importance of playing to citizens’ strengths and utilising their skills and knowhow as well. Professionals highlighted that to make the engaging of citizens in organisational processes and projects more mainstream it was important for interventions to address professionals’ interests too.

#### Citizens

Community-led initiatives described how they were planning to keep (other) citizens engaged throughout initiatives’ phases. For example, Region D’s community-led initiative were looking to change the initiative’s governance structure in such a way that only those people interested in governance matters would be tasked with related issues. They were considering creating a combined and integrated governance structure with other community-led initiatives in the area *(context)*. In this way, they hoped to free up citizens’ time and help them to focus only on the things that interested them, which would help citizens feel more connected to the initiative *(mechanism)*. Ultimately, they expected improved collaboration between the different community-led initiatives in the area *(outcome)*.

#### Professionals

Many professionals had experienced a mismatch between citizens’ and organisational motivations and expectations. Region D PPI professionals described one example where the municipality had stopped providing financial support to a community-led initiative as they felt it was not meeting its goals and was not attracting enough volunteers *(context)*. The citizens were upset that the municipality had pulled their funding and support and so decided to go ahead with the initiative without the municipality *(mechanism)*. However, when local elections loomed, local councillors became interested in the initiative again as they hoped to win more votes *(mechanism)*. Residents remained sceptical of local councillors’ motivations *(outcome)*. Region B’s PPI organisation emphasised that organisations should be transparent about their own motivations and goals behind CE interventions and ensure that these align with citizens’ expectations and interests to enable longer-term collaboration between citizens and professionals.

### Develop a shared CE vision with clear roles for professionals and citizens, ensuring communities’ diversity is reflected within the vision

The authors generated a new principle based on participants’ experiences of implementing CE approaches. The new principle should be seen as the foundation for the others and it underscores that organisations and communities should first develop a shared vision for CE and agree on clear roles for professionals and citizens alike.. However, the majority of the initial CE approaches had been implemented without a clear vision and both citizens and professionals experienced this as a significant barrier to the further development and implementation of CE in their regions. The findings highlight that both citizens and professionals were searching for an underlying vision and were struggling to (a) involve more citizens and communities (i.e. more diversity); (b) to highlight what the shared aims should be; and (c) to define what roles professionals and citizens should have in a more decentralized system.

#### Citizens

Region F’s community-led initiative had already achieved its original aims and were in the process of deciding what their next steps should be. To date, the initiative had only been able to engage citizens who already had an affinity with health and wellbeing, generally white and middle-class residents *(context)*. Some of the volunteers felt that this made it harder for them to ‘unearth’ what other groups’ needs might be, which they felt prevented them from motivating and empowering others to get involved *(mechanism)*. They wanted the initiative to better reflect Region F’s diversity so that healthcare provision would address all residents’ needs *(outcome)*. In contrast, other members wanted to maintain the initiative’s original goal as they felt that engaging disadvantaged citizens would extend their remit and thus formalise and institutionalise the initiative’s structure.

*“One of the initiatives’ biggest success factors was that the village had ownership of the initiative. But now that we’re looking to expand our remit further, it’s a risk too. If you go too far, if you take on too much responsibility, do you not then become just another institute? The initiative is not an institute and it has a different dynamic which you want to keep”* (Citizens)

#### Professionals

Most professionals felt that their roles should be adapted in order to better support communities and their initiatives, but were unclear on what these new roles should entail. Municipal professionals indicated they struggled to find a balance between maintaining municipal accountability (e.g. for services’ quality and safety standards) and enabling citizens to take ownership of projects *(See* Additional file 2*)*. Region D’s PPI professionals pointed out that for municipalities to take on a more supportive role, they would first need to recognise and trust that citizens have more insight, than professionals, into what would improve neighbourhoods and often have the skills and knowhow to implement the improvements too.

## Discussion

Using the realist methodology, this qualitative study investigated how CE is being developed and implemented in six different regions in the Netherlands by examining the contextual factors, mechanisms and outcomes underlying citizens’ and professionals’ definitions and experiences of CE. The study suggests that there are important differences between how citizens and professionals define and experience CE and, relatedly, that organisations and communities were exploring how to adapt to the changes brought on by a newly decentralised system and the related ‘participation society’—e.g. by examining how to effectively adjust their roles and skills. The findings, which resonated with the local reference panel and examined citizens’ and professionals’ experiences in six different Dutch regions and highlighted enablers and barriers to CE.

The findings suggest that developing a shared CE vision, the new principle, is the foundation upon which meaningful CE rest. Participants’ experiences of implementing CE approaches without first discussing an overarching vision of what CE should look like in a more participative and decentralised system, show that without a vision, CE approaches as a whole, and citizens’ roles specifically, remain more limited. For example, within this study, especially municipal professionals described how CE sometimes felt like an imposition; often because local councillors wanted citizens to be engaged without having a clear idea of what the ‘added value’ would be. Professionals implied that municipalities and local councillors feel pressured to engage communities ostensibly because participation has become a key municipal task as reflected within the Participation Act (2015) and the Living Environment Act (expected 2021); but do not necessarily belief CE could help improve policies or projects, or even more broadly local democracy. Ultimately, not having an overarching vision enables organisations to deploy CE to legitimize decisions that they had already taken themselves, rather than including communities more fully in the decision-making process [15].

This study provides additional insight into constraining factors, which should be considered and reflected on if CE is to develop further. One of the most important barriers is that, often fragmented, organisational structures and cultures, and professional remits remain rooted in more traditional models where organisations’ interests are seen as key, rather than citizens’—findings which resonate with previous studies such as [15, 24, 36]. Relatedly, the findings highlight discrepancies between the roles that professionals wish to assign engaged citizens and community-led initiatives, and the new roles and tasks that communities themselves want to take on. As mentioned above, and in line with previous literature like Boviard [4], citizens and community-led initiatives often wish to extend their roles and place them more firmly within the public sphere—e.g. reducing socio-economic health disparities, commissioning services themselves—whereas many organisations still want to contain citizens’ roles within the personal sphere (and use citizens as ‘lay expert’). Professionals’ and citizens’ opposing goals and expectations may lead to tensions between organisations and community-led initiatives if such differences and discrepancies are not openly recognised and discussed. In contrast to the abovementioned barriers, the generating of quick wins in order to build momentum among citizens is an important theme within the literature—and was therefore one of the guiding principles [12]—but was only briefly touched upon by this study’s participants. This is likely because participants had not yet generated quick wins and because organisationally-led CE approaches focused on organisations’ needs, rather than citizens’ needs. Previous studies indicated that quick wins acted as an important enabler, especially in communities where previous CE approaches had failed or in communities with pressing and visible socio-economic needs (e.g. deteriorating infrastructure). Achieving tangible and quick wins can provide momentum and energy for a wider-range of citizens to work together towards other common and achievable goals [13, 16, 49].

The study also highlighted enablers. Previous literature highlighted the importance of empowering and training engaged citizens to ensure they have the right skills and capacity to meaningfully take part in CE approaches (e.g. [10, 11, 13]). However, the professionals within this study emphasised that they themselves also need to develop a new skill-set. Professionals mentioned that to help them adapt their roles to suit meaningful CE, they need (re) training in e.g. how to engage and include a wider range of citizens during earlier project stages. PPI organisations, such as the ones included in this study, could play an important role in upskilling professionals. Furthermore, professionals suggested that such (re) training could help change their organisations’ cultures and help them to provide more supportive and transparent leadership to engaged citizens and community-led initiatives. Such culture changes are necessary to help community-led initiatives and engaged citizens to flourish in the roles they wish to take on themselves. Finally, both already engaged citizens and professionals highlighted the need to take the time to build relationships with harder-to-reach, not yet engaged citizens. The findings, and previous studies like Schoch-Spana et al. [40], suggest that the best way to build such relationships and trust is by ‘going door-to-door’ and attending activities and meetings scheduled by the groups themselves (thus exiting the organisational sphere). Such investments in the building of relationships and changing of organisational cultures and professional skills will be needed if regions are to address power imbalances between organisations and share control more openly with citizens—and develop more inclusive CE visions.

### Future studies

Firstly, future studies will be needed to investigate the implications of a lack of diversity in CE and how best to address such inequalities. The majority of engaged citizens in the six regions were white and middle-class; suggesting current CE approaches are not representative of communities’ heterogeneous nature and differing needs. This raises important questions, including who decides who gets to participate in a ‘participation society’ and whether current CE approaches are in fact further emphasising inequalities in communities and in local healthcare systems. To start addressing such questions, the third stage of this study will be focussing on successful ways to reach out and involve harder-to-reach citizens to enable such groups to be meaningfully engaged as well. Secondly, this study investigated how citizens’ and professionals’ experiences and perceptions were positively or negatively affected by different contextual factors or mechanisms, however, to further develop the evidence-base on CE approaches, future studies could investigate and quantify the actual performance and results of different CE approaches (e.g. regarding health outcomes, changes in policies or service delivery).

### Study limitations

One limitation is the fact that the majority of participants were professionals (42), rather than citizens (15). Additionally, few citizens included in the study belonged to harder-to-reach groups, e.g. low-income households, frail elderly. This limitation is related to how CE is developing in the regions and that most of the CE approaches are organisationally-led and/or operate on the ‘consultation’ and ‘communication’ levels (and therefore include more professionals than citizens). Relatedly, participants were recruited through the local reference panel members’ networks, and because most professionals and engaged-citizens are still searching for ways to reach out to (other) citizens, the number of engaged-citizens was still limited. The authors mitigated this limitation by conducting observations of CE activities (e.g. team meetings of community-led initiatives) so that citizens’ (observed) experiences could be incorporated through fieldnotes. Moreover, the third stage of the study will investigate how vulnerable and disadvantaged groups may more meaningfully engage in the study’s regions.

## Conclusions

By investigating the contextual factors and mechanisms underlying citizens’ and professionals’ definitions and experiences of CE, this study examined how CE is being shaped in six different Dutch regions. The study indicates that citizens and professionals define and experience CE differently and that both organisations and communities were still searching for new visions and roles to suit a newly decentralised system and the ‘participation society’. The findings suggest that in order to further develop CE, organisations and citizens should develop a shared vision of CE with clearly agreed roles and remits. Furthermore, the study suggests that to change organisational cultures and address power imbalances between citizens and professionals, professionals require CE training. This would help ensure that shared visions do not in fact further entrench power differentials. Finally, professionals and already-engaged citizens will need to take active measures to make CE more inclusive and representative of harder-to-reach groups to ensure CE is more diverse and representative.

## Supplementary information

**Additional file 1.** Sample Size.

**Additional file 2.** Summary of Context-Mechanism-Outcome configurations underpinning guiding principles.

## Data Availability

All data generated and analysed during this study are included in the published article and supplementary information files. Templates used for data extraction and analysis are available from the corresponding author on reasonable request.

## Abbreviations

CE: Community engagement
CMO: Context-mechanism-outcome configuration
PPI: Patient & public involvement
