# Supplementary material for: Searching for new community engagement approaches in the Netherlands: a realist qualitative study
Source: BMC Public Health. 2020 Apr 16;20:508. doi: 10.1186/s12889-020-08616-6 (PMC7164336; doi:10.1186/s12889-020-08616-6)
Supplement: Supplementary file 1 — Additional file 1. Sample Size. [file 12889_2020_8616_MOESM1_ESM.docx]

**Appendix 1: Sample Size**

| *Region A* | - One focus group with five professionals from a local municipality (policymakers), from the region’s public health organisation, from a PPI organisation and from a consultancy for small & medium businesses (SMEs) - One interview with a programme-manager (from a primary care support consultancy) |
| --- | --- |
| *Region B* | - One focus group with 12 professionals from a PPI organisation - One focus group with 10 senior-level directors representing seven different sectors (e.g. hospitals, health care insurance companies, general practices) and one engaged citizen who participates in the region’s cross-sectoral governance board - One observation of a CE activity (i.e. board meeting with engaged citizen) |
| *Region C* | - One focus group with six professionals from a local municipality (including e.g. policymakers, communication professionals, community workers) - One observation of a CE activity (i.e. initial scoping exercise to discuss if and how residents wanted to be involved in the development of youth-care policies) |
| *Region D* | - One focus group with three local municipality professionals and two citizens involved in a community-led initiative - An interview with a citizen involved in a Client Council - A joined-interview with a professional from a local hospital and from a local PPI organisation - An interview with one professional from a primary care group - One observation of a CE activity (i.e. workshop between citizens engaged in different community-led initiatives) - One observation of a broader network meeting, including an engaged citizen |
| *Region E* | - One focus group with a local municipality professional, a programme-manager (based at a primary care support consultancy), and one engaged citizen - One focus group with three engaged citizens - One observation of a CE activity (i.e. meeting of the citizen cooperative) |
| *Region F* | - One focus group with eight citizens involved in a community-led initiative - One observation of a CE activity (i.e. workshop and meeting of the citizens involved in the initiative) |
