# Supplementary material for: Searching for new community engagement approaches in the Netherlands: a realist qualitative study
Source: BMC Public Health. 2020 Apr 16;20:508. doi: 10.1186/s12889-020-08616-6 (PMC7164336; doi:10.1186/s12889-020-08616-6)
Supplement: Supplementary file 2 — Additional file 2. Summary of Context-Mechanism-Outcome configurations underpinning guiding principles. [file 12889_2020_8616_MOESM2_ESM.docx]

**Additional file 2: Summary of Context-Mechanism-Outcome configurations underpinning guiding principles**

| ***Guiding principle 1: Citizens’ and professionals’ leadership roles needed to support CE*** | | | | |
| --- | --- | --- | --- | --- |
|  | **Region** | **Contexts** | **Mechanisms** | **Outcomes** |
|  | 1. Region E (citizens) 2. Region E (professionals) | 1. When Cooperative was first established by professionals, the Cooperative were members of cross-sectoral governance board representing the local healthcare system 2. Region E’s professionals struggled to engage citizens other than the ‘usual suspects’ (already engaged citizens) | 1. However, professionals on the board decided to separate the Cooperative from the governance board, which led to the Cooperative feeling disconnected; partly because professionals no longer shared up to date information with them 2. Professionals hoped that the ‘usual suspects’ would want to involve citizens outside their own personal networks and motivate others to get engaged as well. However, professionals were reluctant to impose their own view of CE on citizens | 1. Cooperative missed clear points of connection to professionals in the local healthcare system. Without information about the latest developments, the Cooperative were unable to ensure developments reflected policyholders’ best interests 2. Professionals were still searching for a leadership style that balances supporting the usual suspects to engage other, harder to reach groups and ensuring initiatives’ ownership remained with citizens |
| ***Guiding principle 2: Creating safe & trusting environments*** | | | | |
|  | **Region** | **Contexts** | **Mechanisms** | **Outcomes** |
|  | 1. Region D (citizen) 2. Region B (professional) | 1. Most professionals involved in Region D’s cross-sectoral network kept approaching CE from their own professional mind-set (e.g. planning meetings during office hours, using LinkedIn instead of Facebook to connect with citizens) 2. GP practices in Region B had tried to engage patients by setting up steering groups including both patients and healthcare professionals | 1. Instead, professionals should consider how to make the Network more accessible for citizens (e.g. adjusting meeting times to suit citizens’ timetables, having food served) as taking such small steps to meet citizens’ needs would be appreciated 2. However, many patients in the steering group did not feel safe enough to share their experiences and ideas with the professionals who also provided their care | 1. This might motivate citizens to join and take part in the Network 2. To solve this, the professional felt the practices would need to set up two separate steering groups, one for the patients and one for the healthcare professionals. However, implementing twice the amount of steering groups was thought to be too resource intensive and difficult to scale up. The board decided to invest in training healthcare professionals to be more patient-centred instead |
| ***Guiding principle 3: Citizens’ early involvement*** | | | | |
|  | **Region** | **Contexts** | **Mechanisms** | **Outcomes** |
|  | 1. Region D (professional) | 1. During implementation phase professionals are still trying to wrap their heads around the project | 1. When they are ready to start sharing their ideas or start implementing the project, it is easier and quicker for professionals to approach a colleague or their manager than a citizen. Citizens often feel ‘further removed’ from a professional than their colleagues | 1. She felt that early involvement would therefore be easier if citizens and professionals had clear-points of connection, e.g. weekly meetings |
| ***Guiding principle 4: Shifting decision-making control from organisations to communities*** | | | | |
|  | **Region** | **Contexts** | **Mechanisms** | **Outcomes** |
|  | 1. Region D (citizen) 2. Region D (citizen) 3. Region B (professional) | 1. Currently engaged citizens in the region are usually white and middle-class 2. Community-led initiative struggling to get the other residents interested in more formal and institutional matters (like commissioning) 3. Many projects are not funded without at least one citizen or patient on the project team, so often organisations include just the one citizen or patient | 1. Diversity in CE interventions is necessary to ensure everyone feels able to participate and empower each other 2. They felt it was important not to force residents to take on any institutional responsibilities and to motivate them, instead, to take on such roles by highlighting what the benefits might be for them 3. Professionals then only pay lip service to citizens’ involvement—e.g. by having ad hoc meetings without the citizen—as they were only motivated by the financial implications of the citizen’s involvement | 1. CE can then shape the community and ensure it is more inclusive for everyone 2. Ultimately, the initiative was still searching for ways to balance the dynamic of a community-led initiative while at the same time taking over institutional roles so the village could control its own fate 3. This makes it impossible to establish true partnership between citizens and professionals |
| ***Guiding principle 5: Acknowledging and addressing power imbalances between citizens and professionals*** | | | | |
|  | **Region** | **Contexts** | **Mechanisms** | **Outcomes** |
|  | 1. Region D (citizen) 2. Region D (citizen) 3. Region A (professional) 4. Region B (professional | 1. Citizens are often members because they are service-users, which places them in a more vulnerable position 2. Organisations’ fragmented structures are often a barrier to community-led initiatives’ more holistic views and approaches 3. Perception that only a certain type of citizen were willing and able to participate in community-led initiatives in the rural area (i.e. white & middle-class people who leave the big cities to live out their retirement in Region A) 4. Organisations maintain control over CE, instead of support citizens to take on more ownership themselves | 1. If professionals emphasise service-users’ vulnerability and do not take steps to address service-users’ vulnerable position (e.g. by involving only one service-user in a room full of professionals and keeping meetings within the organisational sphere), then engaged citizens feel patronised and more as service-users rather than full-fledged partners 2. Because municipal departments (e.g. around health and wellbeing, infrastructure) are often fragmented and municipal professionals often do not work collaboratively across departments, community-led initiatives often do not know which processes to follow or which professional to approach and often end up having to establish working relationships with many different professionals, which is demotivating and frustrating for citizens 3. Perception that other citizens, who had lived their whole lives in the region would think already engaged citizens (new Region A residents) would be arrogant and dismissive of their views 4. Because municipalities still feel accountable and responsible for ensuring the projects they finance achieve the agreed upon results. | 1. This emphasises citizens’ dependent position and makes it more difficult to share their opinions and ideas 2. While the community-led initiative understood that every organisation has its structures, they felt professionals should seek internal collaboration between departments in order to better support community-led initiatives’ goals 3. Professionals assumed this was why non-participating citizens did not want to join community-led initiatives. Furthermore this view of already engaged highlighted that Region A’s professionals doubted how representative current initiatives were of everyone’s views. 4. Professionals therefore struggle to share control and place their own expertise as ‘professionals’ above that of citizens as ‘ervaringsdeskundigen’ |
| 1. ***Guiding principle 6: Investing in citizens and professionals who feel they lack the required CE skills or resources*** | | | | |
|  | **Region & intervention** | **Contexts** | **Mechanisms** | **Outcomes** |
|  | 1. Region F (citizens) 2. Region D (citizen) 3. Region D (professional) | 1. Region F’s municipality had not invested financially in or provided support to the community-led initiative 2. Not all citizens are empowered to start engaging without being ‘coaxed’, especially disadvantaged or vulnerable citizens 3. Care Group’s Client Council was still developing and had not yet been integrated within the organisation | 1. Initiative members did not trust the municipality as they suspected the municipality wanted to maintain control of CE within the region. 2. Already engaged citizens should include others in small scale activities (e.g. walking groups) as citizens would feel recognised and appreciated 3. For ‘meaningful’ collaboration, organisations would need to invest in a culture change, because CE still felt like a ‘nice extra’ to professionals rather than part of the organisation’s core business | 1. Due to this mistrust there is no collaboration between the initiative and municipality 2. This way, they can build up their engagement in a manner and at a pace that suits them 3. For the Council to be fully integrated, professionals felt they needed to be offered learning opportunities in how to work collaboratively with the Council and how to integrate the Council more fully |
| ***Guiding principle 7: Quick wins*** | | | | |
|  | **Region** | **Contexts** | **Mechanisms** | **Outcomes** |
|  | 1. Region A (professionals) 2. Region A (professionals) | 1. Previous CE approaches ignored citizens’ input 2. Organisations do not communicate more ‘organisationally-oriented’ results | 1. Citizens often become frustrated with organisations and the CE process if no action is undertaken 2. Professionals feel that organisationally- focused results (e.g. cost-efficiencies) are intangible for citizens and that citizens are uninterested in such results | 1. To prevent this, organisations should communicate clearly what steps are undertaken and/or what results were achieved due to citizens’ input, even if the successes are less tangible for citizens like organisational savings 2. Due to this assumption, such results are not communicated with citizens |
| ***Guiding principle 8: Misaligned motivations*** | | | | |
|  | **Region** | **Contexts** | **Mechanisms** | **Outcomes** |
|  | 1. Region D (professional) 2. Region D (citizens) 3. Region B (professional) | 1. Citizens’ interests vary and therefore want to contribute to different kinds of activities and projects and that they have different time commitments 2. Community-led initiative were hoping to expand their original remit by ensuring the village remained attractive for all age groups, including younger people who often move to bigger cities for employment  - For example, they had approached a local architect to discuss the village’s future housing needs and involved local young people who had previously expressed an interest in the construction sector to take part in the initiative.  1. Service-users tend to only be interested in specific issues related to their own personal experiences | 1. If professionals do not respect citizens’ time commitments or interests, citizens quickly become demotivated and start dropping out of interventions 2. The initiative were hoping to build on residents’ interests, skills and knowhow to motivate them to contribute and get involved. The initiative hoped that the young people themselves would want to help build ten additional houses, which would appeal to their own age group 3. But if specific interests have nothing to do with the issues the CE intervention has been set up to address, service-users and professionals can become frustrated and dissatisfied with the intervention | 1. It is important for professionals to enable residents to decide how and when they would like to participate because only then do citizens remain engaged 2. Ultimately, they wanted more young people to remain in the village, which would in turn also help maintain service provision at its current level 3. With misalignment, citizens and professionals will struggle to collaborate effectively |
|  | ***Guiding Principle 9: Searching for a new vision, a new division of roles, and more diversity to develop CE*** | | | |
|  | **Region** | **Contexts** | **Mechanisms** | **Outcomes** |
|  | 1. Region F (citizen) 2. Region B (professional) 3. Region E (professional) | 1. Cooperative representatives (acknowledging their status as white and middle-class) recognised that the region included lower economic neighbourhoods who they felt whose priority was not to live healthier lives 2. Difficult to engage ‘unorganised’, more vulnerable groups and that CE interventions mostly included ‘professional-volunteers’ who had been on the same Client Council for decades 3. municipality professionals described how she struggled to define her own role when a group of residents approached her with an idea; the citizens wanted to implement a fitness park in their neighbourhood | 1. The Cooperative members felt that a part of their role should be to ‘unearth’ such neighbourhoods’ needs to ensure that the healthcare system not only focussed on those who already had the tools to stay healthy 2. Professionals often questioned the validity of ‘professional-volunteers’ and wondered how representative they were of ‘real’ service-users 3. However, the professional was unsure whether she should just listen to their ideas or take some of the ownership herself to ensure the park would get implemented | 1. They felt that healthcare provision would then be more tailored to such neighbourhoods and eventually wanted to engage the residents within the Cooperative 2. PPI organisation wanted to support organisations to reach out to more vulnerable groups so CE interventions’ structures and processes would reflect their voices too 3. The professional had decided to wait to see whether the citizens themselves could move forward with the project without her help in an attempt to keep ownership with the citizens |
